# Neuroinflammation in dementia with Lewy bodies: a human post-mortem study

**DOI:** 10.1038/s41398-020-00954-8

**Published:** 2020-08-03

**Authors:** Jay Amin, Clive Holmes, Robert B. Dorey, Emanuele Tommasino, Yuri R. Casal, Daisy M. Williams, Charles Dupuy, James A. R. Nicoll, Delphine Boche

**Affiliations:** 1 Clinical Neurosciences, Clinical and Experimental Sciences, Faculty of Medicine, University of Southampton, Southampton, UK; 2 Memory Assessment and Research Centre, Moorgreen Hospital, Southern Health NHS Foundation Trust, Southampton, UK; 3 Department of Cellular Pathology, University Hospital Southampton NHS Foundation Trust, Southampton, UK

**Keywords:** Molecular neuroscience, Physiology

## Abstract

Dementia with Lewy bodies (DLB) is the second most common neurodegenerative cause of dementia, behind Alzheimer’s disease (AD). It is now established that cerebral inflammation has a key role in the aetiology and progression of AD, but this has yet to be confirmed in DLB. We aimed to determine the neuroinflammatory profile in the cerebral cortex of a large cohort of DLB cases. Thirty post-mortem confirmed DLB cases and twenty-nine matched controls were immunolabelled (Brodmann area 21) and quantified for: neuropathology—αSYN, Aβ, P-tau; microglial phenotype—Iba1, HLA-DR, CD68, FcƴR (CD64, CD32a, CD32b, CD16); presence of T lymphocytes—CD3; and anti-inflammatory markers—IL4R, CHI3L1. Status spongiosis, as a marker of neuropil degeneration, was quantified using Haematoxylin and Eosin staining. We found no significant difference between groups in protein load for Iba1, HLA-DR, CD68, CD64, CD32b, IL4R, or CHI3L1, despite increased neuropathology in DLB. CD32a load was significantly lower, and CD16 load higher, in DLB compared with controls. There was no difference in status spongiosis between groups. Significantly more DLB cases than controls showed T-lymphocyte recruitment. Overall, we conclude that microglial activation is not a prominent feature of DLB, and that this may be associated with the relatively modest neuropil degeneration observed in DLB. Our findings, based on the largest post-mortem cohort to date exploring neuroinflammation in DLB, demonstrate a dissociation between protein deposition, neurodegeneration and microglial activation. The relative preservation of cortical structures in DLB suggests the dementia could be more amenable to potential therapies.

## Introduction

Dementia with Lewy bodies (DLB) is the second most common neurodegenerative cause of dementia, behind Alzheimer’s disease (AD)^1^, accounting for 4.2–7.5% of dementia cases^2^. Clinical features of DLB include fluctuating cognition, visual hallucinations and Parkinsonism^3^, with this profile of symptoms leading to people with DLB experiencing a poor prognosis and their caregivers reporting more distress^4,5^. At post-mortem examination of the cerebral cortex, DLB is characterised by the presence of Lewy-related pathology (LRP) in the form of Lewy bodies (LB) and Lewy neurites (LN), formed primarily of alpha-synuclein (αSYN). Amyloid-beta (Aβ) plaques, and to a lesser extent hyperphosphorylated tau (P-tau) tangles, are also present^6^.

Microglia, the innate immune cells of the brain, are highly dynamic cells that display different phenotypes in response to their microenvironment^7^. Whilst in AD neuroinflammation has been widely explored in the form of activated microglia^8^, particularly present around Aβ plaques^9^, the role of inflammation in DLB is yet to be established owing to inconsistent findings. Numbers of activated microglia, immunostained for HLA-DR (Human leukocyte antigen–antigen D related) in post-mortem brain tissue have been shown to be either increased in DLB^10,11^, or unchanged compared with healthy controls^12^. A further study has shown no difference in Iba1 (ionised Calcium-binding adaptor molecule 1) immunostaining of microglia between DLB and controls, but increased Cluster of Differentiation 68 (CD68)-positive microglia in DLB^13^. In addition, the number of activated microglia in the hippocampus (immunolabelled using Iba1 and CD68) were no different in DLB compared with either controls or AD^14^. Lastly, protein immunoreactivity for Iba1 and HLA-DR has been examined in the pulvinar, with no difference found between DLB and controls^15^. In contrast, microglial activation in DLB has been detected in vivo using neuroimaging^16,17^. Overall, there is still no consensus regarding the precise phenotype of microglia in DLB, with most previous studies utilising just one or two markers of microglial activation and in relatively small cohorts.

In Parkinson’s disease (PD), which is closely related to DLB, microglial activation has been demonstrated in the substantia nigra^18,19^, but not in the neocortex^20^. The substantia nigra in PD has also been shown to exhibit CD3+ CD8+ T lymphocyte and immunoglobulin infiltration^21^, whereas CD3 immunoreactivity in the temporal cortex was also detected in a small cohort of DLB cases^22^ and in AD^23^. This supports a role for the peripheral adaptive immune system in neurodegeneration, although it should be noted that one striking pathological feature of DLB is the relative lack of severe neurodegeneration when compared with AD, as observed on structural brain imaging^24^.

Exploration of the relationship between microglial activation, neurodegeneration and adaptive immunity in DLB has the potential to substantially extend our understanding of the aetiology of the disease. In order to characterise the neuroinflammatory profile in DLB, we conducted the largest study to date in this field, using an extensive range of markers of pathology, neurodegeneration and inflammation in human post-mortem brain tissue.

## Materials and methods

### Case selection

Post-mortem brain tissue from 59 donors was obtained from two UK brain banks: Medical Research Council London Neurodegenerative Disease Brain Bank (LNDBB) and the South West Dementia Brain Bank (SWDBB). The middle temporal gyrus (Brodmann area 21) was selected for examination as it is typically burdened with LRP in DLB^6^. All cases were selected following review of case records and post-mortem neuropathological examination reports. DLB cases (*n* = 29) were included if they had a clinical diagnosis as such during life and satisfied neuropathological diagnostic criteria^25^, with a Braak P-tau stage <IV. Control cases (*n* = 30) were selected if they lacked a history of neurological disease and had no significant neuropathology at post-mortem; and were matched for gender, post-mortem delay and age at death. Exclusion criteria for both groups included: <60 years old at death, presence of significant cerebrovascular disease or cerebral amyloid angiopathy, and post-mortem delay >72 hours. Table 1 summarises the baseline characteristics of both groups.

**Table 1:** Demographic and post-mortem characteristics of control and DLB groups.

Variable	Controls (*n* = 29)	DLB (*n* = 30)	*P* value
Age (mean years ±SD)	79.4 ± 8.0	77.6 ± 8.3	0.397^a^
Gender (male:female)	17:12	24:6	0.075^b^
Post-mortem delay (mean hours ±SD)	33.9 ± 15.5	27.3 ± 14.6	0.096^a^
Braak P-tau stage			
Not available	1	3	0.235^b^
0	7	6	
I	6	4	
II	12	8	
III	3	9	
IV–VI	0	0	

Braak P-tau stage unavailable in four cases (three DLB, one control), but neuropathological reports supported no significant co-existing AD pathology. Statistical tests: ^a^independent samples *t* test; ^b^Pearson Chi-squared. Significant results (*P* < 0.05) in italic (none).

### Ethics

NHS Research Ethics Committee approval was provided by LNDBB (London City and East reference 08/H0704/128) and SWDBB (South West Central Bristol reference 08/H0106/28+5).

### Immunohistochemistry

Formalin-fixed, paraffin-embedded 4 µm-thick sections from each case were used for immunohistochemistry. Sections from DLB and control cases were immunolabelled together in batches to allow comparison of staining, with each batch including positive control tissue known to express the protein of interest (e.g., tonsil).

Tissue sections were rehydrated through graded alcohol solutions and water. Endogenous peroxidase was blocked with a 3% hydrogen peroxide solution, followed by heated antigen retrieval and a saturation step to reduce non-specific staining. Sections were then incubated with the relevant primary antibody (Supplementary Table S1) at optimal dilutions. Biotinylated secondary antibodies were obtained from Dako (Glostrup, Denmark) for swine anti-rabbit and rabbit anti-goat antibodies, and from Vector Laboratories (Peterborough, UK) for goat anti-mouse antibody. Binding was visualised using ABC (Vectastain avidin-biotin-peroxidase complex kit, Vector Laboratories, CA, USA) followed by chromogenic reaction with DAB (3,3’-diaminobenzidine peroxidase substrate kit, Vector Laboratories, CA, USA). Counterstaining was performed with haematoxylin to allow visualisation of cellular and tissue structure. All sections were dehydrated and mounted in DePeX (VWR International, Lutterworth, UK).

Neuropathology was assessed with primary antibodies directed against: αSYN (for LRP), Aβ (for plaque pathology) and P-tau (for neurofibrillary tangle and neuritic pathology). Inflammatory proteins were assessed with antibodies to detect: Iba1 (microglial motility^26^), CD68 (lysosomal glycoprotein associated with phagocytosis^27^) and HLA-DR (antigen presentation^27^). FcƴRs, involved in immunoglobulin-mediated immune responses, were examined using CD64, CD32a, CD32b and CD16^28,29^. Anti-inflammatory proteins CHI3L1 and IL4R^30,31^, and the pan-T-lymphocyte marker CD3^32^, were also examined. A set of slides for each case was stained with Haematoxylin and Eosin (H&E) to examine status spongiosis.

### Quantification

Quantification for all pathological and inflammatory markers was assessed in the same anatomical region of interest, blind to group. The Olympus dotSlide digital image capture system (Olympus, Hamburg) was used to capture 30 images at ×20 magnification in a “zigzag” pattern along the grey matter to ensure consistent sampling of all cortical layers, as previously published^33^. Digital images of immunostained tissue were quantified using ImageJ (http://rsbweb.nih.gov/ij/). A specific image threshold was determined for each antibody to quantify the area fraction positively stained, expressed as percentage protein load. The mean of these values was considered representative of the percentage protein load for each marker in each case. Status spongiosis was quantified as the percentage of tissue area that was unstained by H&E, providing an indicator of cortical neuropil degeneration, as previously reported^34^.

**Fig. 1:**
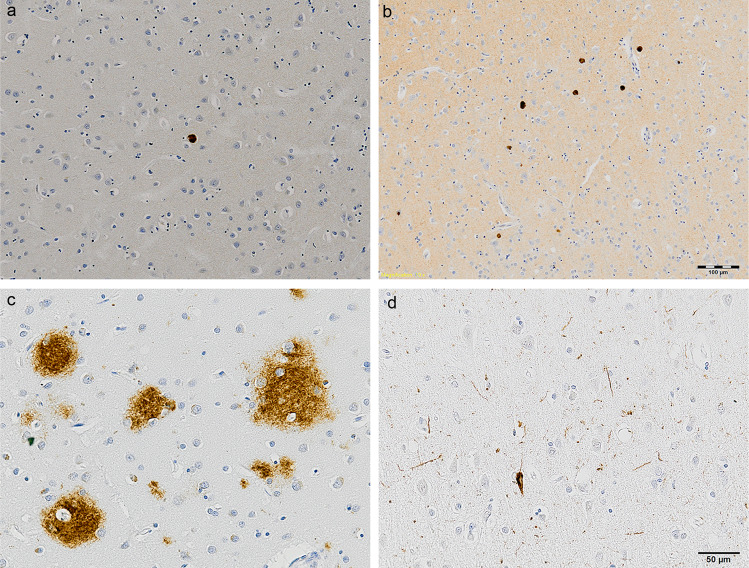
Immunostaining of neuropathology in DLB. Lewy-related pathology in cases scored semi-quantitatively as 1 **a** and 4 **b**. Aβ immunostaining of diffuse plaques **c**. P-tau immunostaining of intra-cellular tangle and neuropil threads **d**. Haematoxylin counterstaining. Scale bar = 100 µm (top row, ×10 magnification) and 50 µm (bottom row, ×20 magnification).

Quantification of αSYN immunostaining was performed using semi-quantitative assessment as recommended by the international consensus diagnostic criteria for neuropathological diagnosis of DLB^25^. The same region of interest as above was assessed using a light microscope (Nikon Eclipse 50i) with ×4 objective lens. The severity of LRP present in each case was graded on a five-point scale, using the following criteria: 0 = none, 1 = mild (sparse LB or LNs), 2 = moderate (more than one LB and sparse LN), 3 = severe (four or more LB and scattered LN), 4 = very severe (numerous LB and LN), as illustrated in Fig. 1.

Immunohistochemistry for the pan-T cell marker CD3 was used to investigate T-lymphocyte recruitment. Semi-quantitative analysis was performed manually upon inspection of the whole tissue section under ×10 magnification using a light microscope. CD3+ T lymphocytes were classified as either present or absent in the parenchyma and perivascular region for both grey and white matter.

### Statistical analysis

Data for continuous outcome variables were assessed for normality of distribution. Baseline characteristics were tested for significant differences using the independent samples *t* test for age and post-mortem delay, and the Pearson Chi-squared test for gender and Braak P-tau stage. Group differences in αSYN severity were determined by the Pearson Chi-squared test. All protein loads (Aβ, P-tau, Iba1, CD68, HLA-DR, CD64, CD32a, CD32b, CD16, IL4R and CHI3L1) were deemed to be non-normal in distribution and the Mann–Whitney *U* test used to compare protein load between DLB and control groups. Spearman rank correlation was used to test for significant associations between markers of neuropathology and inflammation. The Pearson Chi-squared test was used for comparisons of presence or absence of CD3-positive cells between groups in the parenchyma and perivascular areas for grey and white matter. For status spongiosis, the Independent samples *t* test was used for comparisons between groups.

Statistical tests were performed using SPSS (IBM Statistical Package for Social Sciences v24). *P* values of < 0.05 were considered statistically significant for comparisons between DLB and control groups, whereas *P* < 0.01 was deemed significant for correlations between markers within each group to allow for multiple testing.

## Results

**Fig. 2:**
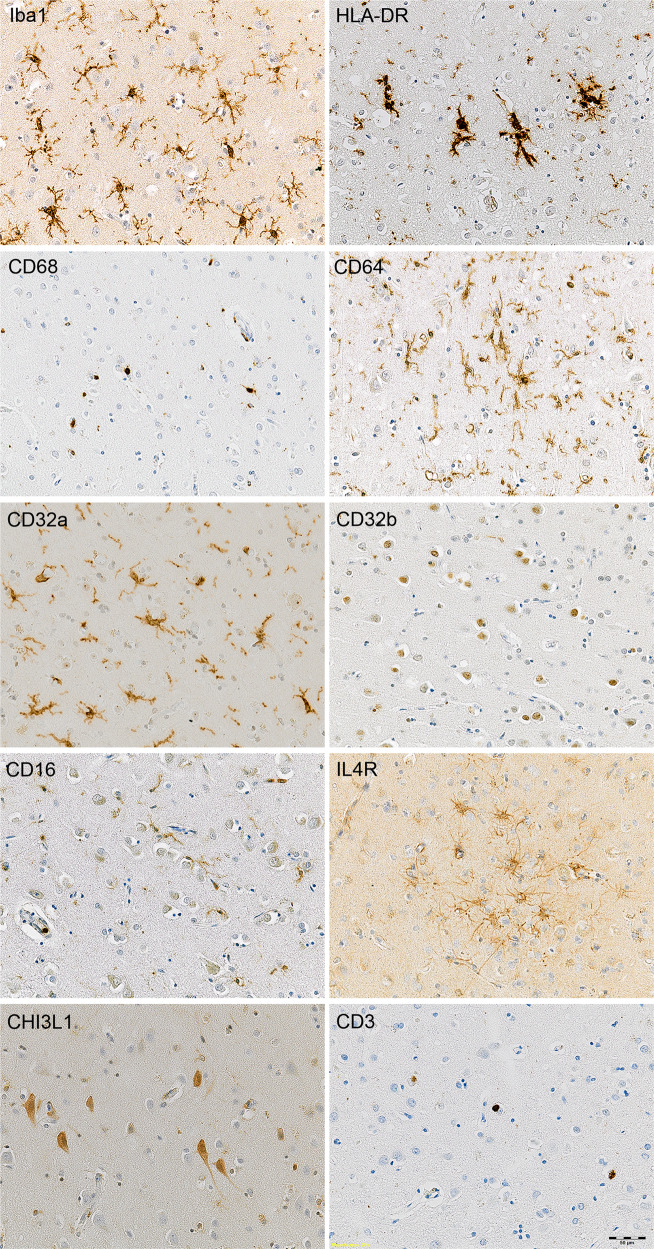
Illustrations of markers of inflammation and CD_3_ T lymphocyte immunolabelling in DLB. Haematoxylin counterstaining. Scale bar = 50 µm (×20 magnification).

Baseline characteristics for the DLB and control groups showed no difference in age, gender, post-mortem delay or Braak P-tau stage (Table 1). A selection of representative digital images of immunostained tissue from DLB cases, showing pictures taken at ×10 or ×20 magnification of grey matter, are presented in Fig. 1 and Fig. 2.

### Neuropathology

Immunostaining for αSYN detected a variable degree of LB and LN in deep cortical layers in the DLB group (Figs. 1a and 1b), with no specific αSYN-positive structures observed in control cases. Aβ immunostaining in DLB was primarily localised to diffuse plaques (Fig. 1c) with sparse dense cores, in addition to limited staining in blood vessel walls; whereas in control cases staining was overall less prominent and localised to diffuse plaque pathology and intraneural staining. P-tau staining was minimal in both DLB and control groups (Fig. 1d).

Quantification of neuropathology revealed significantly higher LRP (*P* < 0.001), Aβ load (*P* = 0.039) and P-tau load (*P* = 0.031) in DLB compared with control cases. Status spongiosis, used as a marker of neuropil degeneration, was not significantly different between DLB and control cases (*P* = 0.614). Data from quantification of neuropathology and status spongiosis are presented in Table 2.

**Table 2:** Quantification of neuropathology, neuropil degeneration, inflammatory markers and CD3+ T lymphocytes.

Marker	Controls (*n* = 29)	DLB (*n* = 30)	*P* value
Lewy-related pathology (LRP) score			
0 – None	29/29 (100%)	3/30 (10.0%)	*<0.001*^b^
I – Mild	0/29	10/30 (33.3%)	
2 – Moderate	0/29	5/30 (16.7%)	
3 – Severe	0/29	11/30 (36.7%)	
4 – Very severe	0/29	1/30 (3.3%)	
Aβ	0.543 (0.119–0.802)	0.914 (0.263–1.496)	*0.039*^c^
P-tau	0.023 (0.013–0.047)	0.042 (0.019–0.068)	*0.031*^c^
H&E status spongiosis	13.73 ± 6.68	12.94 ± 5.28	0.614^a^
Iba1	0.931 (0.408–2.037)	1.128 (0.803–1.694)	0.537^c^
HLA-DR	0.325 (0.249–1.669)	0.436 (0.227–1.146)	0.943^c^
CD68	0.075 (0.051–0.139)	0.116 (0.060–0.223)	0.118^c^
CD64	2.482 (1.722–2.880)	2.580 (1.616–3.309)	0.582^c^
CD32a	0.491 (0.139–0.901)	0.184 (0.086–0.578)	*0.043*^c^
CD32b	0.076 (0.033–0.123)	0.074 (0.031–0.211)	0.705^c^
CD16	0.091 (0.059–0.159)	0.146 (0.098–0.283)	*0.027*^c^
CHI3L1	0.571 (0.238–0.823)	0.344 (0.225–0.695)	0.276^c^
IL4R	0.411 (0.171–0.854)	0.332 (0.174–0.616)	0.912^c^
CD3+ T lymphocytes grey matter parenchyma	14/29 (48.3%)	24/30 (80.0%)	*0.011*^b^
CD3+ T lymphocytes white matter parenchyma	6/27 (22.2%)	16/28 (57.1%)	*0.008*^b^
CD3+ T lymphocytes grey matter perivascular regions	23/29 (79.3%)	21/29 (72.4%)	0.539^b^
CD3+ T lymphocytes white matter perivascular regions	17/27 (63.0%)	22/28 (78.6%)	0.203^b^

LRP scored 0–4 semi-quantitatively, as per consensus diagnostic criteria, and presented as number of cases in each subgroup. Aβ and P-tau presented as median percent protein load (LQ-UQ). H&E status spongiosis presented as mean area fraction unstained ± standard deviation. Percent protein loads for Iba1, HLA-DR, CD68, CD64, CD32a, CD32b, CD16, CHI3L1 and IL4R presented as median (LQ-UQ).

CD3+ lymphocyte data shown as number of cases positive for CD3+ immunolabelling. Statistical tests: ^a^independent samples *t* test; ^b^Pearson Chi-squared; ^c^Mann–Whitney *U* test. Significant results (*P* < 0.05) in italic.

### Inflammatory markers

Immunohistochemistry allowed detection of the following structures, as illustrated in Fig. 2: Iba1, HLA-DR, CD68, CD32a and CD64 showed extensive immunolabelling of microglia and perivascular macrophages; CD32a immunolabelled microglial processes but not cell bodies; CD32b was expressed as neuronal nuclear staining; CD16 immunolabelled microglial cells but also localised to monocytes within vessel lumens; IL4R immunolabelled a sub-population of astrocytes including subpial astrocytes; and CHI3L1 immunolabelled neuronal cytoplasm and some microglial cells.

Quantification of protein load of inflammatory markers, as summarised in Table 2, revealed CD32a load was significantly lower in DLB compared with controls (Control median 0.491% *cf*. DLB median 0.184%, *P* = 0.043), whereas CD16 load was significantly higher in DLB (control median 0.091% *cf*. DLB median 0.146, *P* = 0.027). There was no significant difference in protein load between DLB and control groups for Iba1 (*P* = 0.537), HLA-DR (*P* = 0.943), CD68 (*P* = 0.118), CD64 (*P* = 0.582), CD32b (*P* = 0.705), CHI3L1 (*P* = 0.276), or IL4R (*P* = 0.912).

### T lymphocytes

Analysis of CD3+ immunolabelling demonstrated a significantly higher proportion of DLB cases with T-lymphocyte recruitment to the parenchyma compared with control cases, in both grey (Control cases 14/29 *cf*. DLB cases 24/30, *P* = 0.011) and white matter (control cases 6/27 *cf*. DLB cases 16/28, *P* = 0.008). When assessing for the presence of CD3+ cells in perivascular spaces, there was no difference between DLB and control groups in either the grey (*P* = 0.539) or white matter (*P* = 0.203). These data are summarised in Table 2.

### Relationship between neuropathology and neuroinflammation

Correlation analysis was used to test for significant associations between markers of neuropathology and inflammation. In both control and DLB groups there were no significant associations detected between the markers of neuropathology with any marker of inflammation, as shown in Supplementary Table S2.

We then explored whether neuroinflammatory markers were related within each group. In the control group, significant positive correlations were detected between the following markers: CD68 with HLA-DR (*P* = 0.005), CD64 (*P* < 0.001) and CD16 (*P* = 0.004); CD32a with Iba1 (*P* < 0.001) and CD32b (*P* = 0.003); and CD16 with CD64 (*P* = 0.002). In the DLB group there were significant positive correlations between the following markers: CD68 with CD64 (*P* = 0.002), CD16 (*P* = 0.007) and CD32b (*P* = 0.004); CD32a with Iba1 (*P* = 0.001); CD16 with CD64 (*P* < 0.001); CD68 with CD32b (*P* = 0.004) and IL4R (*P* = 0.001); and HLA-DR with IL4R (*P* < 0.001). These data are summarised in Table 3 for control and DLB groups.

**Table 3:** Correlation between inflammatory markers in control and DLB groups.

	Marker		Iba1	HLA-DR	CD68	CD64	CD32a	CD32b	CD16	CHI3L1
**Controls**	HLA-DR	R_S_	−0.104							
		*P* value	0.591							
	CD68	R_S_	0.047	0.505*						
		*P* value	0.809	0.005*						
	CD64	R_S_	−0.265	0.346	0.616*					
		*P* value	0.173	0.071	0.000*					
	CD32a	R_S_	0.649*	0.229	0.144	−0.075				
		*P* value	0.000*	0.261	0.484	0.722				
	CD32b	R_S_	0.114	−0.118	0.051	−0.108	0.604*			
		*P* value	0.597	0.582	0.813	0.623	0.003*			
	CD16	R_S_	−0.095	0.171	0.522*	0.570*	0.039	0.243		
		*P* value	0.625	0.375	0.004*	0.002*	0.850	0.253		
	CHI3L1	R_S_	0.400	−0.099	0.243	−0.071	0.348	0.282	0.216	
		*P* value	0.032	0.609	0.204	0.718	0.081	0.183	0.260	
	IL4R	R_S_	0.210	0.035	0.003	−0.109	0.376	0.119	−0.107	0.153
		*P* value	0.275	0.858	0.988	0.582	0.059	0.580	0.579	0.427
**DLB**	HLA-DR	R_S_	−0.219							
		*P* value	0.253							
	CD68	R_S_	−0.201	0.399						
		*P* value	0.296	0.029						
	CD64	R_S_	−0.122	0.226	0.550*					
		*P* value	0.538	0.238	0.002*					
	CD32a	R_S_	0.604*	0.128	0.103	0.265				
		*P* value	0.001*	0.516	0.603	0.182				
	CD32b	R_S_	−0.011	0.084	0.540*	0.501*	0.346			
		*P* value	0.957	0.676	0.004*	0.009*	0.083			
	CD16	R_S_	−0.277	0.360	0.481*	0.648*	0.051	0.325		
		*P* value	0.146	0.051	0.007*	0.000*	0.796	0.098		
	CHI3L1	R_S_	−0.208	−0.275	0.008	0.188	−0.227	−0.098	−0.012	
		*P* value	0.279	0.142	0.967	0.330	0.246	0.625	0.949	
	IL4R	R_S_	−0.028	0.682*	0.617*	0.514	0.165	0.456	0.308	−0.117
		*P* value	0.897	0.000*	0.001*	0.010	0.453	0.029	0.135	0.578

Spearman’s rank correlations were conducted to assess for associations between inflammatory markers in the control (top) and DLB (bottom) groups. Values shown are Spearman’s rank correlation co-efficient above *P* values. Significant results (*P* < 0.01) in italic text and marked with an asterisk.

## Discussion

This study utilised the largest cohort of post-mortem DLB cases used to investigate neuroinflammation to date, with an extensive range of inflammatory markers, to define microglial immunophenotype in the cerebral cortex in one of the most common causes of dementia. Intriguingly, we found DLB to be characterised by a lack of neuroinflammation and neuropil degeneration, despite presence of increased neuropathology. We also demonstrated increased cerebral recruitment of T lymphocytes in DLB, supporting a role for adaptive immunity in the disease. The use of post-mortem human brain tissue is a strength of this study as it allows investigation of neuroinflammation in the context of complex human pathophysiology.

In addition to the strengths outlined above, we recognise the following limitations. As with any post-mortem study, we are unable to draw conclusions about the role of inflammation in DLB longitudinally. Microglial activation has been reported in early DLB using in vivo brain imaging^16,17^, raising the possibility that neuroinflammation may peak in mild disease before fading. Another potential limitation relates to the variables of age and gender as potential confounders, although these were mitigated by ensuring that cases were well matched and sourced from the same brain banks. One area worthy of discussion is the inclusion of a single neocortical area in this study. Evidence from studies in PD have shown that LRP may progress sequentially from the midbrain to the neocortex^35^, although it should be noted that this pattern may be markedly different in DLB or mixed DLB/AD cases^36^. Therefore, the presence of neuropathology in one part of the neocortex may not represent the severity of overall neuropathology, but instead the stage of spread. Related to this, we accept that the extent and location of microglial activation may be distinct in different brain regions. However, the examination of both neuropathology and a range of outcome measures in the same brain area allowed us to infer whether protein accumulation was associated with neurodegeneration, microglial activation and recruitment of peripheral adaptive immune cells.

### Neurodegeneration

Structural brain imaging has shown that cortical atrophy is less prominent in DLB than in AD^24^, suggesting that the severity of neurodegeneration may be less severe in DLB. There is also little evidence of extensive loss of cortical synapses in DLB without the presence of concurrent pathology more characteristic of AD^37^. In support of this, our results have shown that the severity of status spongiosis, which is representative of neuropil degeneration in post-mortem brain tissue, was largely unchanged in DLB compared with controls. The differential severity of neurodegeneration between AD and DLB may be associated with differences in the degree of phagocytic phenotype of microglia. Specifically, the strongly phagocytic phenotype of microglia in AD is likely to be linked to higher levels of bystander damage to healthy neuropil.

### Microglial phenotype in DLB

A range of markers previously used to examine activation of microglia in AD were examined in DLB, each purportedly associated with a specific microglial phenotype. Iba1, HLA-DR and CD68 have been most commonly utilised to investigate activation of microglia^30,38^. Iba1 is a microglial cell surface marker associated with cell motility^26,39^, HLA-DR is a marker of antigen presentation^18^ and CD68 a marker of phagocytosis localised to lysosomes^30^.

Our study has confirmed and extended previous work showing an absence of prominent microglial activation in DLB. Specifically, several previous studies have failed to show a significant alteration in the expression of Iba1^13,14^ or HLA-DR^12,15^ in DLB compared with controls, with one study particularly comparable to our work utilising the same Iba1 antibody, method of quantification and brain area^13^. However, two previous studies appear to contradict our findings, both reporting increased HLA-DR-positive microglia in DLB^10,11^. These studies may have been limited by issues relating to study design, with one failing to clearly exclude mixed AD/DLB cases^10^, and the other study only examining a very small area of the brain (five ×200 magnification fields of sections of the hippocampus, amygdala and transentorhinal cortex) in a limited number of DLB cases (*n* = 5)^11^. In PD, activation of cortical microglia is not a prominent feature^20^, whereas transcriptomic analysis does not indicate microglial activation in either DLB^15,40^ or PD dementia^40^. Although it is challenging to compare microglial activation between DLB and PD, especially as LRP is localised differently, it does appear that consensus is building regarding a lack of neuroinflammation in DLB at post-mortem.

Our findings in DLB contrast with data from AD, where increased markers of microglial activation have been consistently demonstrated^38^. Despite being the pathological hallmark of the disease, LRP severity was noted to be relatively modest in our region of interest even in the most severe DLB cases, especially when compared with the dense pathological load found in the AD brain. It has been proposed that the primary pathogenic feature in DLB is not LRP but in fact pre-synaptic αSYN aggregates, which may cause neurochemical imbalance and synaptic dysfunction^41^, but perhaps not the neuroinflammatory profile observed in AD.

Our study confirmed overlap of neuropathology more typical of AD in our DLB group^6^, despite exclusion of mixed cases. However, the severity of Aβ and P-tau pathology present in our DLB cases, whilst greater than that found in the control group, appeared much less prominent than what is typically found in AD. It may be that prominent Aβ and/or P-tau neuropathology is required to drive neuroinflammation in DLB. This theory is supported by previous evidence showing increased temporal lobe neuronal loss in DLB cases possessing prominent AD pathology^42^, and other work that has described worsening cognitive decline in DLB cases that possess severe concomitant AD pathology^6^. Furthermore, the severity of AD pathology found in DLB has been shown to be detrimental to prognosis in a robust and prospectively recruited post-mortem study^43^. Overall, it can be hypothesised that the burden of pathology more typically associated with AD is a key factor in the progression of neurodegeneration in DLB, and probably a driver of any neuroinflammatory response.

### FcγR profile

The expression of FcγR in human post-mortem brain tissue has not been previously reported in DLB. These receptors play a key role in the activation of microglia and co-ordination of phagocytosis, including release of pro-inflammatory cytokines^30^. Human microglia express these receptors at very low concentrations normally, but cerebral levels of the activating FcγR are known to be increased in AD^44^, especially around Aβ plaques^45^. In addition, increased CD64 expression has previously been identified in PD^21^. CD64 is known to have high affinity for IgG (including monomeric IgG), whereas CD32a and CD16 are low affinity receptors for IgG but have high affinity for immune complexes.

The overall balance of activating (CD64, CD32a and CD16) versus inhibitory (CD32b) FcγR on the microglial cell surface dictates the activation level of the cell, and thus the strength of microglial response to immunoglobulin or immune complexes^28^. Our study showed increased protein expression of CD16 and lower CD32a in DLB. The overall result of these changes, particularly in the absence of a difference in CD64 expression, may reflect a compensatory mechanism with an overall unchanged balance of antibody-mediated microglial activation in DLB.

### Anti-inflammatory markers

Neither of the two anti-inflammatory markers included in this study (IL4R and CHI3L1) have been previously examined in the DLB brain. IL4R is a receptor for the anti-inflammatory cytokine IL4, which is known to induce alternative activation of microglia^31^. In the periphery, IL4 is produced by cells of the adaptive immune system, leading to the development and maintenance of wound healing macrophages^46^. CHI3L1, also known as YKL40, is also a marker of alternative activation of microglia^31^.

The expression of both IL4R and CHI3L1 were unchanged in our DLB cases compared with the control group. Both markers have been found to be elevated previously in AD, associated with an immunosuppressive environment in late-stage disease^44,47^. Our study seems to indicate that the anti-inflammatory profile reported in AD is not present in DLB, with this finding consistent with an overall lack of microglial response in DLB and the absence of severe neurodegeneration.

### T lymphocytes

Our study has confirmed increased presence of CD3+ T lymphocytes in DLB, as previously published in human brain tissue in DLB^22^ and PD^48^. We have shown that T lymphocytes were identified in perivascular areas of both control and DLB cases at similar levels, but there was increased infiltration of T lymphocytes into the parenchyma in DLB. Of note, these cells were identified more frequently in both grey and white matter in DLB, implying that their presence is likely to be independent of neuropathology. Involvement of the peripheral adaptive immune system supports the theory that there is crosstalk with the cerebral innate immune system. Increased presence of parenchymal T lymphocytes in DLB may be linked to our finding of increased CD16 expression, which is also expressed on natural killer cells. This combined profile could be associated with the presence of a chronic viral infection. Further work is required to ascertain the specificity of these CD3+ T lymphocytes, and whether they are associated with a role for natural killer cells.

### Associations between neuropathology and inflammatory markers

There were no significant associations between markers of inflammation with neuropathology in DLB. This may be explained by the relative lack of neurodegeneration in DLB limiting the availability of LRP and P-tau to interact with, and activate, microglia. Alternatively, the process of protein deposition in DLB may not induce an immune response owing to the intra-cellular localisation of αSYN, which contrasts with the extra-cellular neuritic plaque pathology that characterises AD.

Associations detected between inflammatory markers in the control group indicate a level of co-ordination of microglial phenotype in response to stimuli, including: phagocytosis (CD68) with antigen presentation (HLA-DR), motility (Iba1) and immune response (activating FcγRs). In addition, the relationship between CD32a and CD32b supports the presence of an equilibrium between activating and inhibitory FcγR in the healthy brain^28^. Overall, these associations reflect co-ordination of inflammation and homoeostasis in healthy conditions to avoid bystander damage. Interestingly in DLB, the pattern of associations between inflammatory markers was altered. The correlations between antigen presentation (HLA-DR) and phagocytosis (CD68), and between CD32a and CD32b, were both lost. In addition, the inhibitory CD32b correlated positively with CD68 and CD64, both markers of a phagocytic phenotype. The anti-inflammatory IL4R was also related with both CD68 and HLA-DR. The DLB brain shows relationships between phagocytic and anti-inflammatory markers, supporting a hypothesis that in DLB microglia showing a more prominent phagocytic phenotype are associated with an increased anti-inflammatory response, both via the inhibitory CD32b and via IL4R, a phenomenon that may be neuroprotective in DLB.

## Conclusions

The role of microglia in DLB has been under-investigated to date with conflicting results. Our post-mortem study supports that DLB is characterised by a lack of microglial activation, which is striking when contrasted with the pronounced phagocytic phenotype of microglia found in AD. This difference may be driven by several factors, including increased neuropathological load in AD, a relative lack of neuropil degeneration in DLB and different genetic risk factors. Of note, we also confirmed the involvement of peripheral adaptive immunity in DLB. Examining the brain after death as part of a retrospective observational study means that is only possible to assess inflammation towards the terminal stage of disease. Whilst important conclusions can be drawn from data at this time point, it is not possible to exclude the possibility of a dynamic inflammatory profile that shifts with disease progression.

The cerebral inflammatory phenotype in DLB is different to that found in AD, and this has important therapeutic implications in suggesting that the current strategies to modulate inflammation as a prevention or treatment for AD may need to be adapted for DLB. Perhaps most intriguingly, the relative lack of cortical degeneration in DLB, with preservation of neuronal machinery being consistent with the typically fluctuating nature of brain function, suggests that DLB may be more amenable than AD to potential therapies.

## Supplementary information

Supplementary Table S1

Supplementary Table S2

## Data Availability

Data that support the findings of this study are available from the University of Southampton repository (10.5258/SOTON/D1019) upon request to the corresponding author.
