# Supplementary material for: Neuroinflammation in dementia with Lewy bodies: a human post-mortem study
Source: Transl Psychiatry. 2020 Aug 3;10:267. doi: 10.1038/s41398-020-00954-8 (PMC7400566; doi:10.1038/s41398-020-00954-8)
Supplement: Supplementary file 1 — Supplementary Table S1 [file 41398_2020_954_MOESM1_ESM.docx]

## Table S1: Primary anti-human antibody characteristics

| **Primary antibody** | **Species** | **Clone** | **Manufacturer** |
| --- | --- | --- | --- |
| αSYN | Mouse | KM51 | Novocastra |
| Aβ | Mouse | 4G8 | Biolegend |
| P-tau | Mouse | AT8 | ThermoFisher Scientific |
| CD3 | Rabbit | IR503 | Dako |
| Iba1 | Rabbit | 019-19741 | Alpha Laboratories |
| HLA-DR | Mouse | M0755 | Dako |
| CD68 | Mouse | M0876 | Dako |
| CD64 | Goat | AF1257 | R&D systems |
| CD32a | Mouse | AB194937 | Abcam |
| CD16 | Goat | AF1597 | R&D systems |
| CHI3L1 | Goat | AF2599 | R&D systems |
| IL4R | Rabbit | HPA050124 | Sigma-Aldrich |

## Abbreviations: α-SYN, Alpha-synuclein; Aβ, Amyloid-beta; P-tau, Hyperphosphorylated tau; Iba1, Ionized Calcium-binding adapter molecule 1; CD, Cluster of Differentiation; HLA-DR, Human leukocyte antigen – antigen D related; CHI3L1 Chitinase-3-like-1; IL4R, Interleukin 4 receptor.
