# Supplementary material for: Neuroinflammation in dementia with Lewy bodies: a human post-mortem study
Source: Transl Psychiatry. 2020 Aug 3;10:267. doi: 10.1038/s41398-020-00954-8 (PMC7400566; doi:10.1038/s41398-020-00954-8)
Supplement: Supplementary file 2 — Supplementary Table S2 [file 41398_2020_954_MOESM2_ESM.docx]

## Table S2: Lack of association between markers of neuropathology and inflammatory markers in Controls and DLB

| **Marker** | | **Controls** | | **DLB** | | |
| --- | --- | --- | --- | --- | --- | --- |
|  |  | **Aβ** | **P-tau** | **α-SYN** | **Aβ** | **P-tau** |
| **Aβ** | R_S_ |  |  | 0.410 |  |  |
|  | P value |  |  | 0.024 |  |  |
| **P-tau** | R_S_ | 0.136 |  | 0.284 | 0.352 |  |
|  | P value | 0.482 |  | 0.135 | 0.061 |  |
| **Iba1** | R_S_ | 0.294 | 0.147 | -0.058 | -0.198 | -0.050 |
|  | P value | 0.121 | 0.446 | 0.764 | 0.303 | 0.800 |
| **HLA-DR** | R_S_ | -0.380 | -0.308 | 0.262 | -0.050 | 0.257 |
|  | P value | 0.042 | 0.104 | 0.162 | 0.795 | 0.178 |
| **CD68** | R_S_ | 0.083 | -0.171 | 0.205 | 0.091 | 0.376 |
|  | P value | 0.670 | 0.375 | 0.278 | 0.634 | 0.045 |
| **CD64** | R_S_ | -0.210 | -0.420 | 0.018 | 0.168 | 0.219 |
|  | P value | 0.284 | 0.026 | 0.924 | 0.382 | 0.263 |
| **CD32a** | R_S_ | 0.000 | -0.030 | 0.104 | -0.259 | 0.128 |
|  | P value | 0.999 | 0.885 | 0.599 | 0.182 | 0.525 |
| **CD32b** | R_S_ | 0.141 | 0.290 | -0.043 | -0.149 | -0.052 |
|  | P value | 0.511 | 0.170 | 0.833 | 0.457 | 0.799 |
| **CD16** | R_S_ | -0.045 | -0.253 | 0.264 | 0.082 | 0.195 |
|  | P value | 0.817 | 0.186 | 0.158 | 0.668 | 0.311 |
| **CHI3L1** | R_S_ | 0.341 | 0.343 | -0.081 | 0.395 | 0.235 |
|  | P value | 0.070 | 0.068 | 0.669 | 0.031 | 0.220 |
| **IL4R** | R_S_ | -0.188 | 0.121 | 0.125 | 0.028 | 0.381 |
|  | P value | 0.330 | 0.532 | 0.552 | 0.895 | 0.060 |

Results are Spearman’s rank correlation co-efficient above P values. Significant results (P<0.01) in italic (none).
